# Medical Department of Louisville University

**Published:** 1849-11

**Authors:** 


### THE WESTERN JOURNAL.

THIRD SERIES.—VOL. IV.—NO. V

LOUISVILLE, NOVEMBER 1, 1849.

### MEDICAL DEPARTMENT OF LOUISVILLE UNIVERSITY.

The present session of the medical school in this city has opened under the most favorable auspices. A large and highly respectable class is already in attendance, and the prospect is flattering for considerable accessions to it. It is unnecessary for us to say anything of the teachers in this school, who have long been connected with it—the past success of the institution is ample evidence of the character of their labors, and of the appreciation of those labors, by the medical mind of the West. But in a Journal, devoted to the advancement and usefulness of the medical profession, it is proper that something shall be said of the increased means of medical instruction which the trustees of the University have secured to the school.

It is known to our readers that Prof. Bartlett, already well and favorably known to the profession both at home and abroad, has accepted the chair of Theory and Practice in the Louisville medical school. A long and intimate acquaintance with the character of this distinguished teacher enables us to speak understandingly of him, and we feel no kind of hesitation in ranking him among the first in his department of medical teaching in this country. He has been a close and diligent student of his profession in the fields of observation, and his contributions to the literature of his profession, and the estimation in which they are held, are a monument in testimony to his merits beyond anything we can say.

Our veneration for this distinguished philosopher is so great, that, if it were not for the fact, that our views of his position as a teacher are in accordance with the general verdict of the profession, we should suspect that a personal regard for him had something to do with our estimate.—But we have no kind of fear as to the position he will occupy in the mind of his classes as a teacher of the theory and practice of medicine. His literary acquirements, his stores of experience in medicine, and the high cultivation of his ample opportunities are sufficient guarantees of what may reasonably be expected from his connection with the Louisville medical school. He has found a proper field now for the exercise of his abilities, and he will give to his chair a character which it has not hitherto possessed. His profound acquaintance with the principles of pathological anatomy is one of the chief elements in his success as a teacher, and is the foundation of much of our hope in his teaching.

The warm attachment we feel to the profession that has occupied much the largest portion of the term of our manhood, induced us to hail the appointment of Dr. Bartlett, to the chair of Theory and Practice in the Louisville medical school, with a sincere satisfaction, because we felt secure in the belief that it would contribute in an eminent degree to the elevation and usefulness of western medicine. The city authorities have erected a commodious room, in connection with the hospital building, for clinical teaching, and Professor Bartlett will give that department a degree of usefulness and success it has never attained before in the West. Demonstrative teaching of pathological anatomy, and appropriate lectures in clinical medicine are the only means, that we know of, for success in medical teaching, and where they are properly attended to, they must command the confidence of the profession. No amount of eloquence, or of medical reading, can offer any substitute for these means of teaching medical science. A school that is defective in them is deficient in everything that can give character and utility to medical instruction. We feel a peculiar gratification in knowing that the Louisville school is amply secured in the departments of teaching, of which we speak, and for this reason we feel a strong confidence that this institution deserves and will enjoy the respect of the profession of the country. Everything that can in any degree qualify men for the successful practice of the art and science of medicine may be found in the Medical Department of the University of Louisville.

Professor Bartlett has commenced his course of lectures, and we are happy to say that his health, long enfeebled by lead poison, is invigorated to such a degree that his friends entertain the confident hope that he will ultimately get rid of all of the effects of his malady. His manifest improvement agreeably disappointed us, and relieved us of much of the apprehension we had felt respecting his constitution. His health is amply sufficient now for the vigorous performance of his arduous duties.

Professor Benjamin Silliman, Jr., who fills the chair of Chemistry under the new arrangement, is discharging his duties with eminent success. He is a remarkably agreeable, instructive and impressive teacher, and cannot fail to win the confidence and esteem of his classes. No one has a more thorough knowledge of the science of chemistry, and he dispenses the rich stores of his knowledge with singular ability. In the manipulations of chemistry he has no superior.

Dr. Lewis Rogers, Professor of Materia Medica, has barely commenced his career as a teacher, but we entertain no doubt of his success. He enjoys the means of making himself an able instructor in his department, and cannot fail to win his way to distinction. He has won eminence as a practitioner of medicine, and will be equally successful in the teaching department of his profession.

We are gratified that the medical school of Louisville is thus invigorated for its labors, and we shall at all times rejoice in seeing her seize upon everything that will contribute to the elevation and extension of medical science. B.

